# Epidural anesthesia for emergency cesarean section in a woman with Fontan circulation: A case report

**DOI:** 10.1097/MD.0000000000018986

**Published:** 2020-01-24

**Authors:** Pin Wu, Sheng-Mei Zhu, Yong-Xing Yao

**Affiliations:** Department of Anesthesia, First Affiliated Hospital, Zhejiang University School of Medicine, Hangzhou, PR China.

**Keywords:** cesarean section, epidural anesthesia, fontan circulation

## Abstract

**Rationale:**

Anesthetic management of pregnant women with Fontan circulation remains challenging. There are few reports that describe the anesthetic management of cesarean section after Fontan surgery. Here, we present a case of successful epidural anesthesia in a woman with Fontan circulation who required emergency cesarean section.

**Patient concerns:**

A 29-year-old woman at gestational week 28 was scheduled for emergency cesarean section because of fetal distress. Her past medical history was significant for congenital transposition of the great arteries that had been treated by Fontan surgery 26 years earlier. Her postoperative course had been uneventful and she had reached a near normal level of activity with no arrhythmias or thrombotic complications. On presentation, her oxygen saturation was approximately 84% and she had digital clubbing. Arterial blood gas analysis showed a PCO_2_ of 35 mmHg, PO_2_ of 55.5 mmHg, and hemoglobin of 16.3 g/dL. Her blood coagulation parameters were within normal limits except for a high fibrinogen concentration (4.55 g/L).

**Diagnosis:**

The diagnosis was pregnancy requiring emergency cesarean section because of fetal distress.

**Interventions:**

Before anesthesia, a radial artery line was established for continuous measurement of blood pressure. An air pressure pump was placed on the patient's lower limbs and a low-dose dobutamine infusion was started. Next, epidural anesthesia was successfully performed at L2–3. Five milliliters of 2% lidocaine followed by 10 mL of 0.75% ropivacaine were injected. Dobutamine was infused to maintain a target blood pressure of 100–120/60–70 mmHg.

**Outcomes:**

The procedure was uneventful with the patient maintaining a stable heart rate of 80 to 90 beats/min and an oxygen saturation of 90% to 94%. A male infant weighing 840 g was delivered. The Apgar score was 9 at 1 and 5 minutes. The patient was transferred to the intensive care unit for 20 hours of monitoring and discharged 9 days later. The neonate was discharged after 2 months of specialist neonatal treatment.

**Lessons:**

Epidural anesthesia may be used in women with Fontan circulation undergoing emergency cesarean section. Knowledge of the physiology of the heart lesion and that of pregnancy are critical to the outcome.

## 1 Introduction

In 1971, Fontan and Baudet described a surgical technique for successful palliation of patients with tricuspid atresia. Subsequently, this technique has been applied to treat most functional single ventricles.^[1]^ The Fontan procedure has undergone several modifications since its initial description, and the surgical indications have expanded. The surgical outcome and long-term survival rate have also improved.^[2,3]^ However, pregnant women who have undergone Fontan surgery are still likely to need cesarean section.^[4]^ The anesthetic management of pregnant patients with Fontan circulation remains challenging. The success of anesthesia directly affects the prognosis. Saito et al recently reported on a woman with Fontan circulation who underwent emergency cesarean section under general anesthesia,^[5]^ while Mathney and Beilin reported successful use of epidural anesthesia for elective cesarean delivery in a woman in a similar situation.^[6]^ However, there is no detailed information in the literature on whether or not epidural anesthesia is feasible in an emergent situation. Here we describe a successful case of epidural anesthesia for emergency cesarean section in a woman with Fontan circulation.

## 2 Case presentation

This case was reviewed by the Ethical Committee Board of First Affiliated Hospital, Zhejiang University School of Medicine (No: 2018-186) and Informed consent was obtained from the patient for publication of her case. A 29-year-old woman (height 155 cm, weight 56 kg) with a pregnancy of 24 weeks and 5 days was admitted because of a decrease in diastolic umbilical artery blood flow and suspected fetal distress. Her past medical history was significant for congenital heart disease (transposition of the great arteries, a ventricular septal defect, and stenosis of the left ventricular outflow tract) that had been treated by modified Fontan surgery 26 years earlier. After corrective cardiac surgery, the right atrium was connected directly to the pulmonary artery and the right ventricle was combined with the left as a functional single ventricle through a large ventricular septal defect. She had fared well postoperatively with a near normal level of activity (New York Heart Association class I–II) and no arrhythmias or thrombotic complications. Her oxygen saturation was about 84% with a nasal catheter supplying oxygen at a flow rate of 2 L/min. Digital clubbing was noted. Doppler echocardiography showed the complicated congenital aortic transposition of Fontan surgery, that is, right atrial and pulmonary artery bridge blood flow and a large ventricular septal defect suggesting a functional single ventricle, with no significant increase in pulmonary artery pressure. Arterial blood gas analysis showed a CO_2_ partial pressure of 35 mmHg, an O_2_ partial pressure of 55.5 mmHg, and a hemoglobin level of 16.3 g/dL. Her blood coagulation parameters were within normal limits, except for a high fibrinogen level of 4.55 g/L. During her hospital stay, she received intensive monitoring and anticoagulation therapy with low-molecular-weight heparin (LMWH).

At gestational week 28, the patient suddenly developed signs of threatened preterm delivery, and monitoring showed a decreased diastolic umbilical artery blood flow and fetal distress. Given her cardiac status and concern regarding the potential for hemodynamic disturbance during vaginal delivery, the decision was taken to perform an emergent cesarean delivery in a cardiothoracic operating theater with immediate availability of cardiopulmonary bypass.

When transferred to the operating room, she received oxygen at a flow rate of 5 L/min via a mask. A radial artery was cannulated for monitoring of arterial blood pressure (BP) and central venous access was established to measure central venous pressure (CVP). On admission, her BP was 112/64 mmHg, her heart rate was 73 beats/min. and her CVP was 13 mmHg. After an air pressure pump was placed on her lower limbs for intermittent driving of blood, a low-dose dobutamine infusion (1–3 μg/kg min) was initiated. After evaluation, epidural puncture was performed at L2–3. Five milliliters of 2% lidocaine followed by 0.75% ropivacaine (10 mL) were slowly injected into the epidural space. Ten minutes after anesthesia, a male infant weighing 840 g was delivered with 1-minute and 5-minute Apgar scores of 9. After delivery, a low-dose continuous infusion of phenylephrine and alprostadil was administered. Her hemodynamic status was stable throughout the procedure (BP 100–120/60–70 mmHg, heart rate 80–90 beats/min, and CVP 16–17 mmHg). Her respiration was stable at a rate of 16–20 breaths/min via an oxygen mask with an oxygen saturation of 90% to 94%. Postoperatively, she was transferred to the intensive care unit for 20 hours of monitoring and was discharged 9 days later. The neonate was discharged after 2 months of specialist neonatal treatment.

## 3 Discussion

Taking into account the anatomic characteristics of the human pulmonary circulation, Fontan and Baudet devised a surgical technique for successful palliation of patients with tricuspid atresia in 1971.^[1]^ Since then, the surgical outcome and long-term survival of patients with complex congenital heart disease have improved dramatically.^[2,3]^ Nowadays, an increasing number of women are reaching childbearing age post-Fontan repair. These women are usually counseled by their physicians to terminate a pregnancy because of delivery-related factors that cannot be controlled. Despite the progress made in terms of anesthetic technique and the anesthetic agents available, management of women with Fontan circulation in the peripartum period remains challenging for anesthesiologists. A thorough understanding of the physiology of the partially corrected heart lesion and the impact of the physiologic changes of pregnancy is critical to successful management of cesarean section in these women. We have presented a case of successful epidural anesthesia for emergency cesarean section in a woman with Fontan circulation. The operation was uneventful, and both the mother and neonate were discharged after an uneventful postoperative course.

Fontan surgery, also known as a pulmonary collateral procedure, is performed through the right atrium and pulmonary artery. The difference in pressure between the vena cava and left atrium produces a pressure gradient (right atrium - pulmonary artery - pulmonary vein - left atrium) that promotes perfusion of the pulmonary vascular bed with venous blood. The procedure is palliative rather than curative, but in many cases can result in normal or near-normal pump function and body growth. The common complications after a Fontan procedure include heart failure, arrhythmia, and thrombosis.^[7]^ Moreover, pregnant patients with Fontan circulation face volume overload and hypercoagulation, both of which require additional specialist treatment.^[8,9]^ Such factors add to the difficulty of perioperative anesthetic management in these women. With the advent of routine preventive anticoagulation therapy, intravertebral anesthesia (such as epidural block) may no longer be the best choice in emergent situations. However, in the present case, prompt cessation of LMWH paved the way for an epidural block. More than 12 hours had elapsed since the last dose of LMWH and the patient's blood coagulation parameters were within normal range, so epidural block was reconsidered. Nevertheless, to minimize the risk of intravertebral hemorrhage or hematoma, the epidural puncture was performed by an experienced anesthesiologist and LMWH was restarted 12 hours postoperatively.

In the presence of mixed venous blood, severe hypoxia may lead to marginal myocardial function and loss of compensation, resulting in circulatory collapse. The key to preventing lethal hypoxia and stabilizing hemodynamic status in a patient with Fontan circulation is to ensure the pressure gradient between the vena cava and left atrium. General anesthesia provides a secure airway but usually requires mechanical ventilation, which increases the mean intrathoracic pressure and pulmonary vascular resistance and decreases the venous return and pulmonary blood flow, thereby decreasing oxygenation in a patient with Fontan circulation. However, epidural block allows spontaneous breathing, which produces a low intrathoracic pressure and may be a more suitable technique in such patients, even in emergent situations. We performed an epidural block in our patient to ensure stable pulmonary vascular resistance and oxygenation with adequate intraoperative volume load and peripheral vascular resistance. It is also essential to maintain sinus rhythm and myocardial contractility for preservation of cardiac function and to maintain the pressure gradient.^[10]^ Therefore, we used a low-dose dobutamine infusion intraoperatively to maintain a BP of 100–120/60–70 mmHg and a heart rate of 80 to 90 beats/min, thereby increasing hemodynamic performance.

## 4 Conclusions

Epidural block may be helpful in women with Fontan circulation undergoing cesarean section, even in emergent situations. However, a thorough understanding of the physiology of Fontan circulation is essential for successful perioperative management. It is critical to maintain hemodynamic stability and ensure an appropriate BP gradient. Finally, management of such patients requires multidisciplinary collaboration between clinicians who are familiar with congenital heart disease, including cardiologists, obstetricians, and cardiac anesthetists.

## Acknowledgments

We would like to thank Editage (www.editage.com) for English language editing.

## Author contributions

**Conceptualization:** Yong-Xing Yao.

**Writing – original draft:** Pin Wu.

**Writing – review & editing:** Sheng-Mei Zhu.

## References

[1] Fontan F Baudet E Surgical repair of tricuspid atresia. Thorax 1971;26:240–8. 5089489 10.1136/thx.26.3.240 PMC1019078

[2] AboulHosn JA Shavelle DM Castellon Y Fontan operation and the single ventricle. Congenit Heart Dis 2007;2:2–11. 18377510 10.1111/j.1747-0803.2007.00065.x

[3] Tweddell JS Nersesian M Mussatto KA Fontan palliation in the modern era: factors impacting mortality and morbidity. Ann Thorac Surg 2009;88:1291–9. 19766824 10.1016/j.athoracsur.2009.05.076

[4] Cauldwell M Steer PJ Bonner S Retrospective UK multicentre study of the pregnancy outcomes of women with a Fontan repair. Heart 2018;104:401–6. 28954835 10.1136/heartjnl-2017-311763

[5] Saito J Noguchi S Nakai K General anesthetic management for emergency cesarean section and postpartum hemorrhage in a woman with Fontan circulation. J Cardiothorac Vasc Anesth 2019;33:791–5. 29935806 10.1053/j.jvca.2018.05.012

[6] Mathney E Beilin Y Successful epidural anesthesia for cesarean delivery in a woman with Fontan repair. J Clin Anesth 2015;27:60–2. 25468587 10.1016/j.jclinane.2014.08.006

[7] Bonner SJ Asghar O Roberts A Cardiovascular, obstetric and neonatal outcomes in women with previous Fontan repair. Eur J Obstet Gynecol Reprod Biol 2017;219:53–6. 29054041 10.1016/j.ejogrb.2017.10.013

[8] Chugh R Management of pregnancy in women with repaired CHD or after the Fontan procedure. Curr Treat Options Cardiovasc Med 2013;15:646–62. 23893314 10.1007/s11936-013-0263-4

[9] Canobbio MM Warnes CA Aboulhosn J Management of pregnancy in patients with complex congenital heart disease: a scientific statement for healthcare professionals from the american heart association. Circulation 2017;135:e50–87. 28082385 10.1161/CIR.0000000000000458

[10] Oh C Youn JK Han JW Hepatocellular carcinoma after the Fontan procedure in a 16-year-old girl: a case report. Medicine (Baltimore) 2016;95:e4823. 27741102 10.1097/MD.0000000000004823 PMC5072929

